# Longitudinal serological and virological survey of hepatitis E virus in wild boar (*Sus scrofa majori*, Maremman wild boar) and fallow deer (*Dama dama*) populations in a protected area of Central Italy

**DOI:** 10.3389/fvets.2024.1511823

**Published:** 2024-11-27

**Authors:** Luca De Sabato, Mariagiovanna Domanico, Paola De Santis, Daniele Cecca, Giulia Bonella, Giovanni Mastrandrea, Roberta Onorati, Luigi Sorbara, Bianca Maria Varcasia, Barbara Franzetti, Andrea Caprioli, Antonio Battisti, Fabio Ostanello, Ilaria Di Bartolo

**Affiliations:** ^1^Department of Food Safety, Nutrition and Veterinary Public Health, Istituto Superiore di Sanità, Rome, Italy; ^2^Istituto Zooprofilattico Sperimentale del Lazio e della Toscana “M. Aleandri”, Rome, Italy; ^3^Segretariato generale della Presidenza della Repubblica – Servizio Tenuta di Castelporziano, Rome, Italy; ^4^Italian Institute for Environmental Protection and Research ISPRA, Rome, Italy; ^5^Department of Veterinary Medical Sciences, University of Bologna, Bologna, Italy

**Keywords:** hepatitis E, wild boar (*Sus scrofa*), fallow deer (*Dama dama*), infection, genotypes, HEV-3, zoonosis, Italy

## Abstract

Hepatitis E virus (HEV) is recognized as an emerging zoonosis. Pigs and wild boars are considered the main reservoirs of zoonotic HEV-3 and HEV-4 genotypes. In Europe, autochthonous human cases of hepatitis E, mainly associated with HEV-3 and consumption of raw or undercooked pig and wild boar liver/meat, have increased over the last decades. From 2016 to 2024, during several hunting seasons, we conducted a molecular and serological longitudinal survey on the circulation of HEV in Maremman wild boar (Italian subspecies/ecotype, *Sus scrofa majori*) and fallow deer (*Dama dama*) populations in a protected area in Central Italy. During the study period, 346 livers (256 from wild boar, 90 from fallow deer), 161 serum (127 from wild boar, 34 from fallow deer), and 23 meat juice (11 from wild boar, 12 from fallow deer) samples were collected. Serum and meat juice samples were tested using a commercial ELISA test for the detection of total anti-HEV antibodies. An estimated serological prevalence of 28.3% (39/138) in wild boar and 21.7% (10/46) in fallow deer was found. The 346 liver samples were tested using a HEV Real-Time RT-PCR for the detection of HEV-RNA. Thirty-one wild boar (12%) and four fallow deer (4.4%) livers were found positive. Phylogenetic analysis of 11 partial ORF2 sequences from wild boar confirmed the HEV3 heterogeneity in this species, revealing different strains (3f, 3c) circulating over the years. The detected subtypes are among the most commonly detected in Italy and our strains showed a high correlation with human and wild boar Italian strains. Although the studied area is a fenced natural reserve, the presence of different strains over time suggests the probable virus introduction from the external. Our results confirm fallow deer susceptibility to the infection, and that wild boar could be considered the main wild HEV reservoir. This is also the first study demonstrating the infection in the so-called Italian subspecies/ecotype Maremman wild boar. Moreover, our results corroborate that the consumption of undercooked or raw liver from both wild boar and fallow deer, or the direct contact with these animals, could represent a zoonotic risk.

## 1 Introduction

Hepatitis E (HE) is an acute viral disease caused by the hepatitis E virus (HEV) and characterized by the fecal-oral route transmission (1). HEV is a quasi-enveloped single-strand RNA virus classified in the family *Hepeviridae*, genus *Paslahepevirus*, which includes the *Paslahepevirus balayani* species, divided into eight genotypes (2). HEV genotypes have been mainly associated with specific host species and geographical origin (2). The HEV-1 and HEV-2 genotypes, restricted to humans, primarily circulate in endemic areas (Asia and Africa) causing several outbreaks linked to the ingestion of contaminated water. In non-endemic areas, infections by HEV-1 and HEV-2 are related to travel in endemic areas. HEV-3 and HEV-4 genotypes infect humans and several animal species, among which pigs and wild boars are considered the main reservoirs (3). The presence of HEV-3 has been extensively described in pig populations around the world, with high seroprevalence (up to 100%) which increase with age (4, 5).

HEV sequences classified within the HEV-3 genotype are highly variable, and although only one serotype has been identified so far, the observed differences, based on phylogenetic analysis of complete genome sequences and subgenomic regions, allow for the further classification into subtypes, named in alphabetical order. So far, HEV-3 is classified into 13 subtypes (a-o), and novel subtypes are constantly proposed (2, 6).

In the last 20 years, an increasing number of infections have been described linked to the zoonotic transmission of HEV-3 and HEV-4, and these genotypes are now considered endemic in some developed countries (3, 7). Zoonotic HEV-3 and HEV-4 infections in high income countries are mainly linked, by direct or indirect evidence, to the consumption of raw and undercooked pork products (mainly liver sausages) (7) and undercooked wild boar meat (1).

In Europe, including in Italy, HEV-3 is the most frequently detected genotype in humans, pigs and wild boar (4, 8). HEV-4, mainly found in Asia, has also sporadically been detected in Italy in pigs and in one human case (9, 10).

In wild boar, HEV seroprevalences are highly variable ranging between 4.9% (11) and 57.4% (12). Studies on the detection of HEV-RNA in wild boar and other wild ungulates also varies greatly in relation to different specimens tested (feces, liver, bile, blood, and muscle) and geographic areas (7, 13–27). HEV-3 RNA in *Suidae* is most frequently detected in bile and liver, the organ of virus replication, and subsequently in feces (4). Among European countries, HEV-RNA detection in wild boar liver samples also varies widely, ranging between 3% (28) and 68.2% (29). HEV-positive animals can be detected in every age group, including juveniles and adult animals older than 24 months, although prevalence is generally higher in juveniles that are infected following the loss of passive immunity (26, 30).

Heterogeneity of HEV-3 viral strains is higher in wild boar (6), where many subtypes have been detected. The Italian wild boar HEV strains sequenced so far are mainly classified into 3e, -3f, -3c and, less frequently, -3a and -3m subtypes, but of notice that many detected strains could not be subtyped (6, 11, 19, 31–43).

Several cervid species are also known to be susceptible to the infection, with previous investigations across Europe showing variable HEV seroprevalence values, ranging from 0.4 to 19.5%, and HEV-RNA prevalence varying between 1.2 and 34.1% (24, 44–55). Concerning fallow deer, in Italy, only one study reported the presence of HEV-RNA in one out of 60 tested livers (55).

The aim of the present study was to conduct a longitudinal serological and virological survey of HEV in wild boar (*Sus scrofa majori*, Maremman wild boar) and fallow deer (*Dama dama*) populations in a protected area of Central Italy. Detected HEV strains were typed and subtyped by sequencing and were also subjected to phylogenetic analyses to establish correlations with the other circulating viral strains.

## 2 Materials and methods

### 2.1 Sampling and location

Liver, muscle, serum, and meat juice samples were collected from a total of 260 apparently healthy Maremman wild boars and 92 fallow deer during slaughterhouse activities. Overall, 346 livers (256 from wild boars, 90 from fallow deer), 161 serum (127 from wild boars, 34 from fallow deer) and 23 meat juice (11 from wild boars, 12 from fallow deer) samples were collected (Table 1). Wild boars were shot and subsequently sampled in several hunting seasons (from October to January) in the period 2016–2024. Fallow deer were sampled during the period 2018–2023. During 2017 and 2021, samples were not collected due to management problems and due to the occurrence of the SARS-CoV-2 pandemic.

**Table 1:** Samples collected during the years of the study.

Year of sampling	Number of sampled animals	Livers sampled	Sera sampled	Meat juice sampled	Liver and sera samples from the same animal (paired samples)
Wild boar					
2016	53	53	53	0	53
2017	0	0	0	0	0
2018	32	31	32	0	31
2019	26	25	15	0	14
2020	10	8	7	0	5
2021	0	0	0	0	0
2022	42	42	13	5	18
2023	66	66	7	6	13
2024	31	31	0	0	0
Total	260	256	127	11	134
			Total sera + meat juice 138		
Fallow deer					
2018	1	0	1	0	0
2019	49	49	22	0	22
2020	5	4	4	0	3
2021	0	0	0	0	0
2022	25	25	7	8	15
2023	12	12	0	4	4
Total	92	90	34	12	44
			Total sera + meat juice 46		

**Figure 1:**
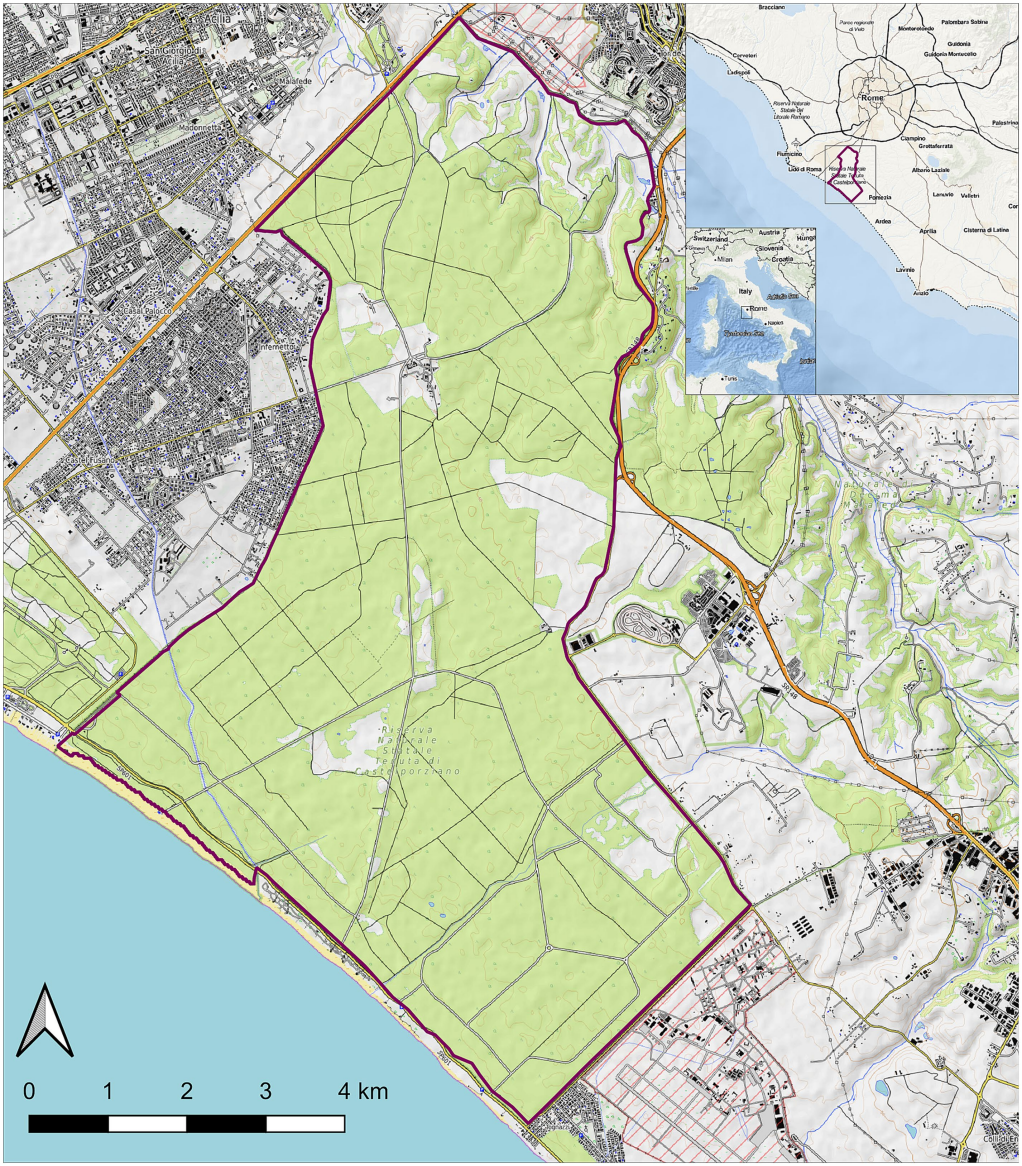
Map of the Castel Porziano Estate, Rome, Italy. At the upper right corner, location within Italy and location within the Latium Region are showed. Red lines represent the borders.

Animals were from the protected area of Castelporziano Presidential Estate (CPE), located in Central Italy at around 25 km from the center of Rome (41°44′037.83″00N; 12°24′02.20″00E) (Figure 1). The CPE includes most of the coastal ecosystems typical of Mediterranean area, consisting of lowland hygrophilous woodlands, especially in proximity of wetlands, featuring evergreen and deciduous oak trees. The CPE hosts numerous animals, including domestic species such as horses and cattle (but not pigs) and wild ungulates species including wild boars, fallow deer, roe deer, and smaller groups of red deer. The entire CPE is fenced and supposedly the animals inside should not have contact with exogenous ungulate populations. In particular, the CPE hosts a wild boar population referable to the so-called endemic Italian wild boar subspecies/ecotype: *Sus scrofa majori* (the Maremman wild boar) (56–58). Ungulates population densities inside the CPE are annually evaluated in fall by distance sampling with thermal imaging and in spring by direct counts on fixed positions. Estimated population densities greatly change during years and seasons and in the last three checked years (2020–2023) resulted of around 30–50 wild boars/km^2^ and 10–15 fallow deer/km^2^, respectively. To control and regulate the ungulate populations, since 1982 fallow deer and wild boar culling campaigns and wild boar trapping campaigns have been carried out in the fall/winter and summer, respectively. Animals are usually shot all around the CPE, collected, and conducted to the slaughterhouse located inside the CPE, where the carcasses are routinely inspected and the meat sent for human consumption.

During slaughtering, along with the samples, demographic data for each animal were also recorded: age, sex, and hunting area. For both species, animals were aged by the evaluation of tooth eruption and replacement patterns and categorized in two classes: young (under 1 year of age) and subadult–adult (over 1 year of age) (Table 2).

**Table 2:** Age and sex of the animals collected during the years of the study.

Year of sampling	Total number of sampled of animals	Sex		Age categorization	
	Wild boar	Male/female		Young/subadult–adult	
2016	53	18	35	11	42
2017	0	0	0	0	0
2018	32	25	7	2	30
2019	26	15	11	11	15
2020	10	7	3	2	8
2021	0	0	0	0	0
2022	42	14	28	14	28
2023	66	32	34	18	48
2024	31	7	24	5	26
Total	260	118	142	63	197
	**Fallow deer**	**Male/female**		**Young/subadult–adult**	
2018	1	0	1	0	1
2019	49	20	29	23	26
2020	5	3	2	4	1
2021	0	0	0	0	0
2022	25	13	12	9	16
2023	12	7	5	6	6
Total	92	43	49	42	50

### 2.2 Detection of anti-HEV antibodies

Briefly, blood was obtained from the heart or thoracic cavity of the sampled animals (*n* = 161) during routine slaughterhouse procedures. Samples were stored at 4°C during transport from slaughterhouse to laboratory. There, the samples were allowed to clot at 4°C overnight and then centrifuged at 1.500×*g* for 10 min. The separated serum samples thus collected were stored at-20°C until further testing. To extract meat juice, skeletal muscle samples (mainly diaphragm) were collected (*n* = 23) using sterile scalpels at the end of routine slaughtering, transported under chilled conditions at 4°C, and stored at −20°C until processing. Samples were thawed overnight at room temperature and mechanically squeezed to obtain 200–500 μL of meat juice. Due to technical issues, serum or meat juice were not obtained from all the sampled animals.

Total anti-HEV antibodies (IgG, IgM, and IgA) in serum and meat juice samples were detected using a double antigens sandwich HEV multispecies ELISA kit (HEV ELISA 4.0v. MP Diagnostics-Biomedicals, Eschwege, Germany), a multispecies kit developed exclusively for veterinary use, following manufacturer’s instructions. Optical density was read at 450 nm using the plate reader Infinite® F50 (Tecan, Männedorf, Switzerland). Following the manufacturer’s instruction, a sample was considered positive when its OD was higher than the threshold defined as the mean OD for negative controls +0.3.

### 2.3 PCR sample preparation and nucleic acid extraction

Liver samples (*n* = 346) were collected (approximately 5 g) using sterile scalpels at the end of the evisceration stage during routine slaughtering. Samples were stored at 4°C during transport from slaughterhouse to laboratory, where they were stored at −80°C until processing. Fifty milligrams of each liver were spiked with 10 μL of a process control virus (Mengovirus strain MC_0_, 1.6 × 10^5^ TDCI_50_/mL), and the samples were homogenized in 650 μL of lysis buffer (RLT) with zirconia beads, using a mechanical disruptor (Tissue Lyser, Qiagen, Milan, Italy). The total RNA was extracted by the RNeasy Mini kit (Qiagen, Milan, Italy) as previously described (5), and immediately analyzed or stored at −80°C until processing. The RNA concentrations were determined using the NanoDrop One (Thermo Fisher Scientific, Wilmington, DE, USA). An OD 260/280 ratio of 1.8–2.1 at pH 7.5 was considered acceptable.

### 2.4 HEV-RNA virus detection and sequencing

HEV and the process control Mengovirus RNA were detected by Real-Time RT-PCR (RT-qPCR) as previously described (5, 59). A water negative control for the extraction procedure, a PCR positive control consisting of viral RNA derived from a HEV positive swine fecal sample, already available in the laboratory, a Non-template control (NTC), and an Internal Positive Control (IPC) were used as controls. The extraction efficiency was estimated by the comparative cycle threshold (Ct) method (60). The RNA samples were considered positive when they showed a Ct value <38. Samples with Ct ≥ 38 were considered negative. If the sample IPC Ct value was higher than the NTC IPC Ct value, the sample was considered inhibited, and an additional 10-fold dilution of the extracted RNA was tested. Extracted RNA from RT-qPCR HEV-positive liver samples was also analyzed by nested RT-PCR amplifying a 348 bp fragment of ORF2 as previously described (61, 62). The obtained DNA amplicons of expected size were sent to the external company Eurofins Genomics (Ebersberg, Germany) to be sequenced. Sequences were deposited in GenBank NCBI database with Accession Numbers PQ283405-PQ283415.

### 2.5 HEV subtyping and phylogenetic analyses

A maximum likelihood phylogenetic tree was built using the sequences obtained in this study (*n* = 11), 19 HEV-3 reference sequences and 119 closest sequences obtained by Blastn searches on NCBI database (accessed on January 2024), selecting those with >91% of nucleotide identity. A HEV-4 strain was used as outgroup (LC022745). Sequences were aligned using Aliview software (63) and the maximum likelihood tree was built using IQ-TREE version 2 (64) with 1,000 bootstrap replicates, using the substitution model suggested by the software after the model test analysis.

### 2.6 Statistical analysis

Univariate analysis was performed to assess factors associated with antibodies positivity and HEV-RNA prevalence in livers considering the sex and age group using the Chi-squared test or Fisher’s Exact Test when cell counts were less than 5. Tests and tables were produced using the R software v. 4.1.2.^1^ Confidence intervals were calculated using the prevalence library in R and the Exact interval (Clopper-Pearson interval).

## 3 Results

### 3.1 Serological results

**Figure 2:**
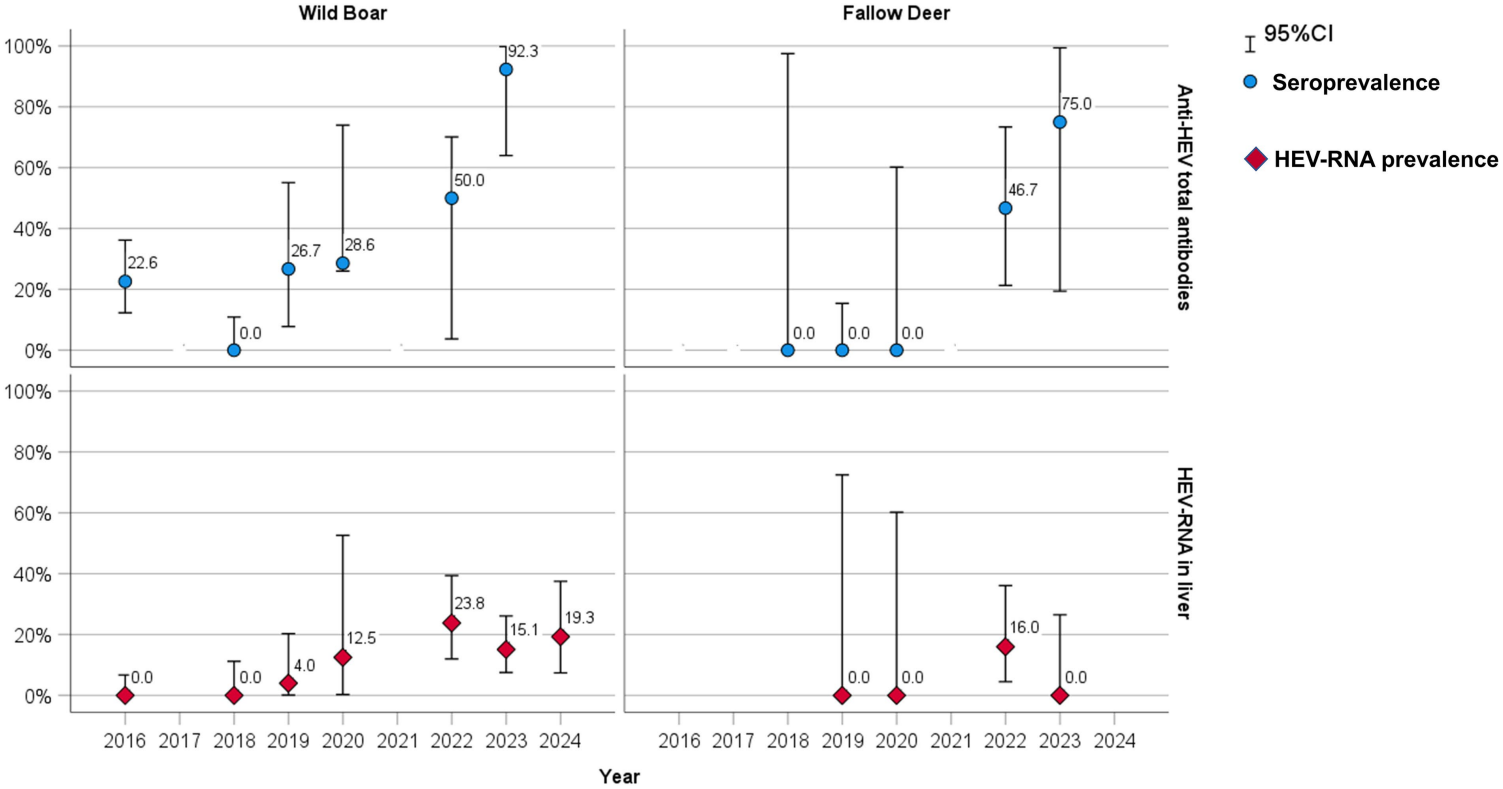
Seroprevalence (blue dots) and RT-qPCR HEV-RNA prevalence (red diamonds) rate by sampling year in wild boar and fallow deer.

A total of 39 out of 138 wild boar serum and meat juice samples, with an overall estimated serological prevalence of 28.3% (95% CI: 21.0–36.5) and a total of 10 out of 46 fallow deer serum and meat juice samples (21.7, 95% CI: 10.9–36.4) tested positive for anti-HEV antibodies throughout the years of the study. In wild boar, the estimated seroprevalence was 16.9% in males (11/65, 95% CI: 8.8–28.3%) and 38.4% (28/73, 95% CI: 27.2–50.5%) in females (*p* < 0.01, X-squared = 6.7695, df = 1). Considering age, 18.5% of young (5/27, 95% CI: 6.3–38.1) and 30.6% of subadult–adult animals (34/111, 95% CI: 22.2–40.0) tested positive (*p* = 0.31, X-squared = 1.0308, df = 1) (Table 3). Seroprevalence varied greatly during the years, ranging from 92.3% (12/13, 95% CI: 64.0–99.8) in 2023 to 0% in 2018 (0/32, 95% CI: 0.0–10.9) (Figure 2).

**Table 3:** Anti-HEV total antibodies detection in wild boar and fallow deer grouped by sex and age class.

		Wild Boar			Fallow deer		
		Positive/Total (%)	95% CI	*p*	Positive/Total (%)	95% CI	*p*
Sex	Male	11/65 (16.9)	8.8–28.3	<0.01	5/22 (22.7)	7.8–45.4	1
	Female	28/73 (38.4)	27.2–50.5		5/24 (20.8)	7.1–42.1	
	Total	39/138 (28.3)	21.0–36.5		10/46 (21.7)	10.9–36.4	
Age class	Young	5/27 (18.5)	6.3–38.1	0.310	5/20 (25.0)	8.7–49.1	0.912
	Subadult–adults	34/111 (30.6)	22.2–40.0		5/26 (19.2)	6.5–39.3	
	Total	39/138 (28.3)	21.0–36.5		10/46 (21.7)	10.9–36.4	

In fallow deer, seroprevalence was 22.7% in males (5/22, 95% CI: 7.8–45.4) and 20.8% (5/24, 95% CI: 7.1–42.1) in females (*p* = 1, X-squared = 1.2646e−30, df = 1). Considering age, 25.0% of young (5/20, 95% CI: 8.7–49.1) and 19.2% of subadult–adult animals (5/26, 95% CI: 6.5–39.3%) tested positive (*p* = 0.912, X-squared = 0.012041, df = 1) (Table 3). Seropositive animals were detected only in 2022 (7/15, 46.7, 95% CI: 21.3–73.4) and in 2023 (3/4, 75, 95% CI: 19.4–99.4), while all the tested sera were negative in 2018, 2019 and 2020 (Figure 2).

### 3.2 RT-qPCR HEV-RNA virus detection in liver samples

A total of 31 out of 256 wild boar liver samples (12.0, 95% CI: 8.4–16.7) and 4 out of 90 fallow deer liver samples (4.4, 95% CI: 1.2–10.1) tested RT-qPCR positive throughout the years. In wild boars, HEV-RNA prevalence was 12.8% in males (15/117, 95% CI: 7.4–20.3%) and 11.5% (16/139, 95% CI: 6.7–18.0%) in females (*p* = 0.898, X-squared = 0.016305, df = 1). Considering age, 20.6% of young (13/63, 95% CI: 11.5–32.7%) and 9.3% of subadult–adult animals (18/193, 95% CI: 5.6–14.4%) tested positive (*p* < 0.05, X-squared = 4.6939, df = 1) (Table 4). HEV-RNA prevalence varied greatly during the years, ranging from 23.8% (10/42, 95% CI: 12.0–39.4) in 2022 to 0% in 2016 and 2018 (Figure 2).

**Table 4:** HEV-RNA virus detection in wild boar and fallow deer liver samples grouped by sex and age class.

		Wild boar		Fallow deer			
		Positive/Total (%)	95% CI	*p*	Positive/Total (%)	95% CI	*p*
Sex	Male	15/117 (12.8)	7.4–20.3	0.898	2/42 (4.8)	0.5–16.1	1
	Female	16/139 (11.5)	6.7–18.0		2/48 (4.2)	0.5–14.2	
	Total	31/256 (12.0)	8.4–16.7		4/90 (4.4)	1.2–10.1	
Age class	Young	13/63 (20.6)	11.5–32.7	<0.05	2/41 (4.8)	0.6–13.4	1
	Subadult–adults	18/193 (9.3)	5.6–14.4		2/49 (4.1)	0.5–13.4	
	Total	31/256 (12.0)	8.4–16.7		4/90 (4.4)	1.2–10.1	

In fallow deer, the HEV-RNA prevalence was 4.8% (2/42, 95% CI: 0.5–16.1) in males and 4.2% (2/48, 95% CI: 0.5–14.2) in females (*p* = 1). Considering age, 4.8% of young (2/41, 95% CI: 0.6–13.4) and 4.1% of subadult–adult animals (2/49, 95% CI: 0.5–13.4) tested positive (*p* = 1) (Table 4). Positive samples were found only in 2022 (4/25, 16, 95% CI: 4.5–36.1), while were all negative in 2019, 2020 and 2023 (Figure 2).

### 3.3 HEV subtyping and phylogenetic analyses

**Figure 3:**
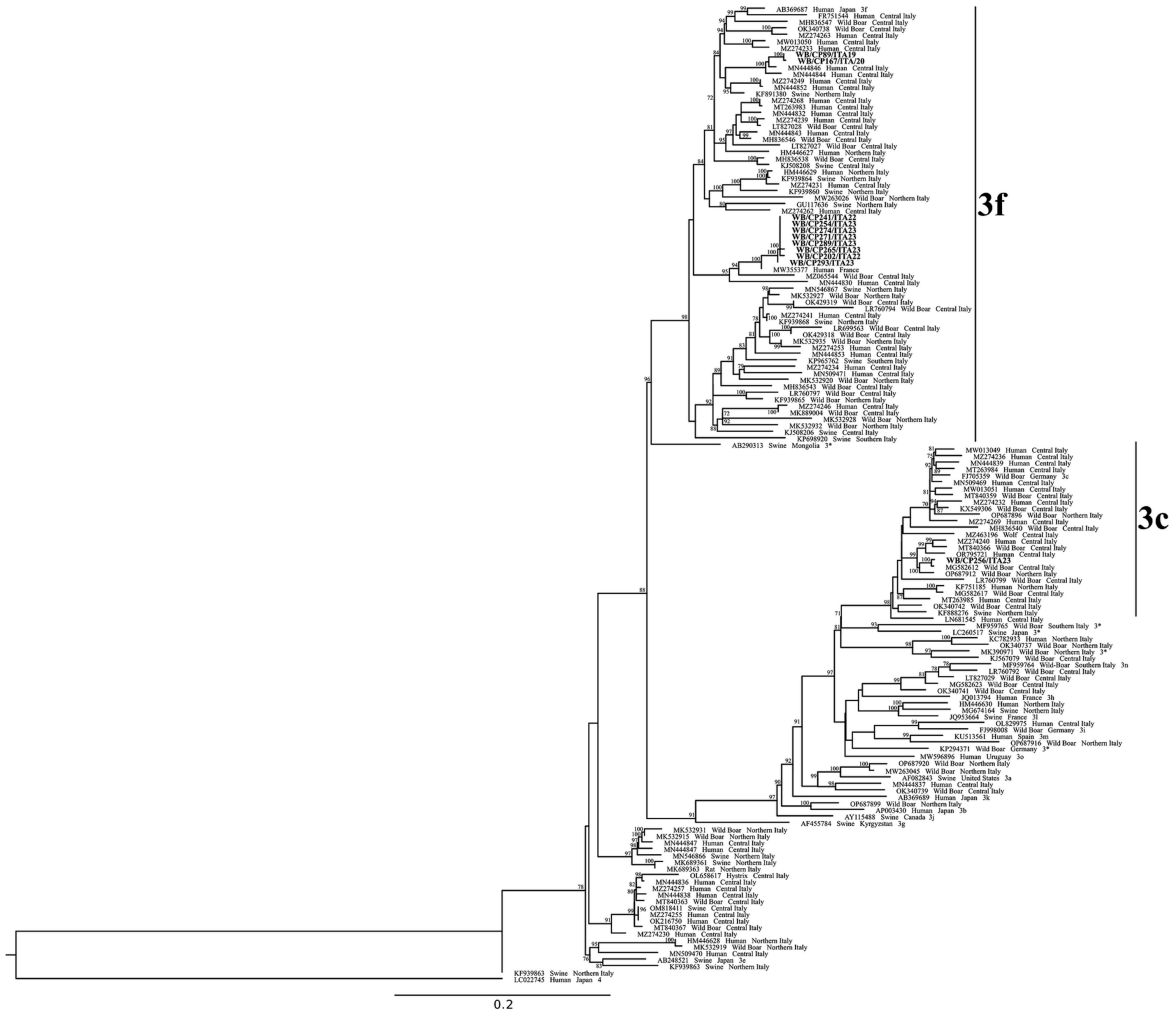
Phylogenetic tree based on 290 nt fragment within ORF2 of 150 HEV sequences: 11 sequences obtained in this study (highlighted in bold), 119 HEV-3 sequences obtained from NBCI database by BLASTn searches, 19 HEV-3 reference sequences and a HEV-4 strain used as outgroup. The maximum likelihood tree was produced using the TN model (Tamura-Nei) with invariant sites and gamma distribution based on 1,000 bootstrap replications. Bootstraps values >70 are indicated at their respective nodes. Sequence entries are reported as GenBank Accession Number, Host species and Country. For reference sequences also the subtype name is reported.

Out of the 35 RT-qPCR positive livers (31 from wild boar and 4 from fallow deer), 11 were also positive by nested RT-PCR and sequenced. All the 11 obtained sequences were from wild boar and belonged to HEV-3 genotype (GenBank Accession Numbers PQ283405-PQ283415). Ten sequences were classified as 3f and one as 3c (Figure 3). Sequences classified as 3f formed two clusters, with a nucleotide identity (nt. id.) <89.0% among each other. The first cluster included two strains from animals hunted in 2019 and 2020 (WB/CP89/ITA19 and WB/CP167/ITA20), sharing 99.4% nt. id. one to each other. The strains in this cluster showed the highest nt.id. (approximately 97.0%) with two Italian strains detected in 2012 and 2015 from human patients hospitalized in Central Italy (INMI_1205_2012, MN444846; INMI_1516_2015, MN444844).

The second one, including eight sequences obtained from animals captured in 2022 and 2023, displayed an intragroup nt. id. between 98.9 and 100% one to each other, with five strain sequences being identical. The strains in this second cluster displayed the highest nt.id. (97.3–98.1%) with a French human strain (HESQL108, MW355377) reported in 2021 (Figure 3).

If compared only with Italian strains, the eight sequences grouped with three other strains: one detected from a wild boar captured in Central Italy in 2021 (91.3–92.2% nt.id.: S87, MZ065544), one detected in 2017 from a HEV human case hospitalized in Central Italy (89.8–90.0% nt. id.: INMI_1712_2017, MN444830), and one from a swine sampled in 2012 in South Italy (88.4–88.9% nucleotide identity: KP698920, SwHEVE14IT12).

The 3c sequence (WB/CP256/ITA23), identified from an animal captured in 2023, formed a cluster with five Italian strains, three reported in wild boars and two from humans. The closest was identified from a wild boar in Central Italy in 2016 (99.0% nt. id.: WB27VT2016, M7G582612), and specifically in an area 200 km apart from the CPE. The other strains (<96.4% nd.id.) were reported in 2019 in wild boars from Northern (92247, OP687912) and Central (2019.AZ.6234.1.1, MT840366) Italy, while the human strains were reported in 2017 (ISS_ID_219/2017, MZ274240) and 2022 (INMI_FG03_2022, OR795721) in Central Italy (Figure 3).

## 4 Discussion

The serological and virological survey on HEV in wild boar and fallow deer we conducted has the value to be a longitudinal study performed during several hunting seasons (2016–2024), with the possibility to study the infection dynamic in a confined restricted area (5.892 hectares), with a high ungulates density. Indeed, the survey was performed in a protected area where hunting game activities were prohibited as early as 1977, and was subjected to conservation measures since 1999. The CPE perimeter is completely fenced, so the animals inside are supposed to have no contact with any domestic pig nor with exogenous ungulate populations. This survey is one of the few HEV studies conducted on fallow deer, and to our knowledge, the only one conducted to the so-called endemic Italian wild boar subspecies/ecotype: *Sus scrofa majori* (the Maremman wild boar), a population with a high genomic differentiation and morphometric uniqueness that could represent the only surviving “pure-bred native stock” in Italy (57, 58). The only other similar population is located in the Maremma Regional Park in Tuscany (56, 57).

In wild boar, we found an overall average HEV seroprevalence of 28.3% and a prevalence of HEV-RNA in liver samples of 12.0%. Our serological and virological results are similar to those obtained in some other studies conducted in Italy in the European wild boar (7, 25, 37, 65, 66). Some other studies reported higher HEV-RNA prevalences: 43.6% (67), 33.5% (34), and 16.3% (39). The differences may be attributed to variations in sample types, assays used across studies, factors linked to the specific wild boar populations, and/or geographic areas. It is also noteworthy that other previous studies were conducted on wild boar populations living in non-confided areas.

As expected, the identified genotype belonged to HEV-3 and we found two different subtypes (3f and 3c), representing the main HEV strains identified in the EU in both humans cases and wild boar (3). Although not statistically significantly, we detected a higher seroprevalence of HEV-specific antibodies in adult wild boar, while the HEV-RNA detection was higher in younger animals. This is in line with the known course of infection, and the development of immunity, as already observed in other studies conducted in wild boars (32, 68) or pigs (4, 5). In this study, we also found a significantly higher seroprevalence in female wild boar than in males, while no significant difference is evident for the RNA detection rates. In suids, the relation of HEV-infection with sex is variable depending on the studies, but in general, a lack of sex related association is reported (24).

In fallow deer, the observed seroprevalence was 21.7%, while HEV-RNA detected in liver samples was 4.4% (RNA positive samples were detected only in 2022). Unfortunately, we could not identify the genotype circulating in fallow deer, probably due to the low RNA available in the samples. In fallow deer, HEV-RNA was detected for the first time in Germany in sera from hunted animals, with a prevalence of 4.3%, close to the one found in our study (48), although in the same country, a recent investigation examining liver samples reported a lower prevalence of 1.19% (24). This latter prevalence is more similar to that reported in a previous study conducted in Central Italy that found the presence of HEV-RNA in one fallow deer liver sample out of 60 tested (1.7%) (55). Other studies conducted on fallow deer in several European countries (Germany, Portugal, and Sweden), did not find any HEV-positive serological or virological samples (49, 53, 69, 70).

The lower HEV-RNA and antibody prevalence we detected in fallow deer, with HEV-RNA positive samples found only in 2022, could indicate a primary circulation in wild boars and only a spillover transmission to fallow deer over time. We can suppose that the strict contact with wild boars, in a close restricted area with high population densities, could have led to possible frequent contacts, and a high level of HEV exposure for fallow deer, resulting in transmission. Further studies are needed to confirm this hypothesis, although similar prevalence and infection dynamic results were previously reported in Germany (24, 49). Nevertheless, descriptions of human cases linked to consumption of raw deer meat have been previously reported (71, 72), confirming the susceptibility of cervids to the infection, and highlighting the importance of implementing control measures to avoid zoonotic transmission from these species.

Regarding the circulation of the infection in both species over time, although no samples were collected in 2017 and 2021, we observed variable serological and virological prevalences over the years of the survey (2016–2024). In wild boar we observed a persistence of the infection and an increasing trend of positivity along the years, with peaks in 2022–2023, while in fallow deer the virus apparently emerged for the first time only in 2022, when the density of wild boar reached a peak (59.2 wild boar/km^2^). This trend could suggest a possible recent virus introduction, or, alternatively, fluctuations of the HEV infection dynamic in the animal populations, due, for instance, to groups of animals with different infection status moving within the area, possible variations in immunity (e.g., for stress factors), etc. Environmental factors such as water sources, food availability, and human activities might also have influenced the HEV transmission, and in the future they could be further investigated.

About the virus characterization, the fact that only 11 wild boar HEV-positive liver samples were confirmed by nested RT-PCR out of the 31 positives by RT-qPCR, is likely linked to the viral titer or RNA amount in the original samples (37), which is a variable related with the analytical sensitivity of the two different methods. The sequence analyses performed in this study, confirmed the heterogeneity of HEV-3 strains in wild boars, revealing different subtypes (3f, 3c) and strains circulating over the years (Figure 3). In particular, we found different 3f strains circulating in 2019–2020 and in 2022–2023. Moreover, in 2023, we found the co-circulation of 3f and 3c subtypes. These results would support the hypothesis of different virus introduction over time. In Italy, the HEV-3f and 3c are among the most common circulating subtypes, the former in both swine/wild boar and humans, and the latter mainly in wild boar (8, 73). In line with subtypes distribution, some sequenced strains reported in this study were highly correlated to Italian sequences reported in both wild boars and humans in Central Italy and in the same region (Lazio region) (8, 17, 62, 73, 74), respectively. Although the studied area is completely fenced, and no introduction of ungulates from outside officially occurred, the presence of different circulating strains over time suggests the probable introduction of the virus from external sources either by direct contact between animals (likely other wild boars from outside), or through contaminated fomites. To be noted that in 2023, inside the CPE, an outbreak of European Brown Hare Syndrome (EBHS) occurred in the resident Italian hare population, likely due to an external introduction of the virus (75). Our results would confirm the possibility for some viruses to overcome the isolation measures and/or the strict biosecurity procedures applied in the estate and more in general in nature reserve areas.

As for similar previous studies (8, 25, 37, 39, 43, 74), the phylogenetic analysis we performed also showed that the wild boar strains we identified are genetically close to other human strains circulating in the same geographical area, with the exception of eight sequences of 3f, more related to a French human strain. Hepatitis E disease is now recognized as a zoonosis of public health concern and growing interest. Humans become infected mainly by eating contaminated undercooked or cured liver or either by contact with infected animals (76). In this respect, the detection of the virus in livers from both wild boars and fallow deer represents a zoonotic risk, also considering the possibility of cross-contamination of other edible parts of the carcass. This is particularly true for Central Italy, where consumption of locally produced wild boar food, specifically cured meat and liver, is a typical local eating habit. Besides, also hunters, slaughterers and other people working with wild ungulates should be considered at risk for contracting the infection, because of routine handling of live animals and carcasses during their activities.

## 5 Conclusion

The longitudinal survey we conducted in a protected and fenced area of Central Italy confirms the spread of HEV in wild ungulates. Our results also confirm the fallow deer susceptibility to the infection, probably as a spillover, and that wild boars could be considered the main wild HEV-reservoir. Many studies on HEV circulation in wild boars were previously conducted in Italy, but this is the first one demonstrating the infection in the so-called wild boar Italian subspecies/ecotype (*Sus scrofa majori*), suggesting that the infection dynamics is similar regardless of the genotypic and phenotypic characteristics of the host. As expected, we identified the HEV-3 genotype, with a variability of circulating subtypes (3c and 3f) and strains. These subtypes are among the most common found in Italy, and our strains were found to be genetically close to other human and wild boar HEV strains circulating in Italy and Europe. Although the studied area was a fenced natural reserve, the presence of different circulating strains over time suggests the probable introduction of the virus from outside, and its ability to spread rapidly to wild ungulates.

Our results also corroborate that the consumption of undercooked or raw liver from both wild boar and fallow deer, or the direct contact with these animals, could represent a zoonotic risk.

The publicly available HEV nucleotide sequences obtained in this study may be useful for comparing present and future human and animal strains, useful for insights into transmission events between wild boar, farmed pigs, and humans. As for other known zoonotic agents, HEV should be included in national or regional wild animal diseases surveillance programs. Further studies are needed to assess the epidemiology, the intra-and inter-species transmission, the maintenance of the HEV infection and strain phylogeny in wild ungulates.

## Data Availability

The datasets presented in this study can be found in online repositories. The names of the repository/repositories and accession number(s) can be found in the article/supplementary material.

## Footnotes

^1^ https://cran.r-project.org
